# Mechanical tibial loading remotely suppresses brain tumors by dopamine-mediated downregulation of CCN4

**DOI:** 10.1038/s41413-021-00144-2

**Published:** 2021-05-24

**Authors:** Yao Fan, Rongrong Zha, Tomohiko Sano, Xinyu Zhao, Shengzhi Liu, Mark D. Woollam, Di Wu, Xun Sun, Kexin Li, Motoki Egi, Fangjia Li, Kazumasa Minami, Amanda P. Siegel, Takashi Horiuchi, Jing Liu, Mangilal Agarwal, Akihiro Sudo, Harikrishna Nakshatri, Bai-Yan Li, Hiroki Yokota

**Affiliations:** 1 Department of Phamacology, School of Pharmacy, Harbin Medical University, Harbin, China; 2 Department of Biomedical Engineering, Indiana University Purdue University Indianapolis, Indianapolis, Indiana USA; 3 Department of Orthopedic Surgery, Mie University, Mie, Japan; 4 Peking Union Medical College Hospital, Chinese Academy of Medical Sciences, Beijing, China; 5 Department of Chemistry and Chemical Biology, Indiana University Purdue University Indianapolis, Indianapolis, IN USA; 6 Integrative Nanosystems Development Institute, Indiana University Purdue University Indianapolis, Indianapolis, IN USA; 7 Graduate School of Engineering, Mie University, Mie, Japan; 8 Department of Physics, Indiana University Purdue University Indianapolis, Indianapolis, IN USA; 9 Department of Radiation Oncology, Osaka University Graduate School of Medicine, Suita, Osaka Japan; 10 Simon Cancer Center, Indiana University School of Medicine, Indianapolis, IN USA; 11 Department of Surgery, Simon Cancer Research Center, Indiana University School of Medicine, Indianapolis, IN USA

**Keywords:** Cancer, Bone quality and biomechanics

## Abstract

Mechanical loading to the bone is known to be beneficial for bone homeostasis and for suppressing tumor-induced osteolysis in the loaded bone. However, whether loading to a weight-bearing hind limb can inhibit distant tumor growth in the brain is unknown. We examined the possibility of bone-to-brain mechanotransduction using a mouse model of a brain tumor by focusing on the response to Lrp5-mediated Wnt signaling and dopamine in tumor cells. The results revealed that loading the tibia with elevated levels of tyrosine hydroxylase, a rate-limiting enzyme in dopamine synthesis, markedly reduced the progression of the brain tumors. The simultaneous application of fluphenazine (FP), an antipsychotic dopamine modulator, enhanced tumor suppression. Dopamine and FP exerted antitumor effects through the dopamine receptors DRD1 and DRD2, respectively. Notably, dopamine downregulated Lrp5 via DRD1 in tumor cells. A cytokine array analysis revealed that the reduction in CCN4 was critical for loading-driven, dopamine-mediated tumor suppression. The silencing of Lrp5 reduced CCN4, and the administration of CCN4 elevated oncogenic genes such as MMP9, Runx2, and Snail. In summary, this study demonstrates that mechanical loading regulates dopaminergic signaling and remotely suppresses brain tumors by inhibiting the Lrp5-CCN4 axis via DRD1, indicating the possibility of developing an adjuvant bone-mediated loading therapy.

## Introduction

Mechanical loading is therapeutically important, particularly in bone homeostasis. Loading to the tibia, for instance, activates canonical Wnt signaling and strengthens the tibia weight-bearing capacity.^1^ This conversion of an outer environmental force into intracellular signaling in bone-forming osteoblasts is orchestrated by force-sensing osteocytes in the bone matrix.^2^ Notably, mechanosensitive osteocytes also exhibit tumor-suppressing ability in breast cancer. We have previously shown in a mouse model that the correct amount of mechanical loading to the tibia suppresses tibial tumor-induced osteolysis.^3^ Whether loading to bone can suppress tumor growth in nonbone tissues in a remote location is unknown. In this study, we investigated the effect of tibial loading on the progression of brain tumors associated with breast cancer.

About one in eight U.S. women develops breast cancer in her lifetime, and invasive cancer preferentially metastasizes to tissues such as bone, liver, lung, and brain.^4,5^ Brain metastasis leads to one of the worst prognoses with an extremely low overall survival rate.^6,7^ While neurosurgery, radiation therapy, and stereotactic radiosurgery are common treatments, these therapies can severely impair neurocognitive ability and quality of life with limited survival benefits.^8,9^ Most chemotherapeutic agents frequently used for the treatment of breast cancer are not fully effective because of the blood–brain barrier.^10^ This study examined the possibility of converting remotely applied mechanical loading into antitumor signaling in the brain.

The primary focus herein was the effect of mechanical loading on dopamine-mediated tumor suppression. We and others have shown that dopamine modulators such as A77636, trifluoperazine, and fluphenazine can reduce the progression of breast cancer via two types of dopamine receptors (DRD1 and DRD2).^11–15^ Furthermore, we have shown that skeletal loading to the mouse hind limb elevates the urinary level of dopamine.^16^ Importantly, dopamine does not cross the blood–brain barrier; thus, intravenous administration does not deliver dopamine to the brain. In dopamine synthesis, tyrosine hydroxylase (TH) serves as the rate-limiting enzyme,^17^ and Erk is reported to regulate the expression of TH.^18^ While electrostimulation has been shown to elevate dopamine levels in the midbrain,^19^ we examined whether loading to the tibia upregulates TH via Erk signaling and promotes dopamine synthesis in the ventral tegmental area (VTA), which includes abundant dopaminergic neurons.^20^ In addition, we sought to determine whether tibial loading induces dopamine-mediated suppression of brain tumors via D1- or D2-type dopamine receptors.

However, the mechanism of remote mechanotransduction from the bone to the brain and the role of Lrp5-mediated Wnt signaling, which is required for loading-driven bone gain, need to be elucidated.^21,22^ However, Lrp5 in cancer cells promotes tumor progression, and many challenges have been overcome to block Wnt signaling for cancer treatment.^23^ Considering the dual role of Wnt signaling in the mechanotransduction of bone and tumor progression, we hypothesized that loading-driven dopamine in the brain downregulates Lrp5 to suppress tumor growth. In response to the loading-driven elevation of dopamine, we evaluated the expression of Lrp5 and downstream effector genes in tumor cells, including tumor-promoting cytokines and oncogenic genes such as Src, Snail, MMP9, Runx2, and TGFβ. A protein array analysis was used to predict cellular communication network factor 4 (CCN4, also known as WNT1-inducible signaling pathway protein 1) as a dopamine-responsive target to be inhibited.^24^

## Results

### Mechanical loading to the tibia upregulated dopamine-synthesizing enzyme in the brain and serum

**Fig. 1:**
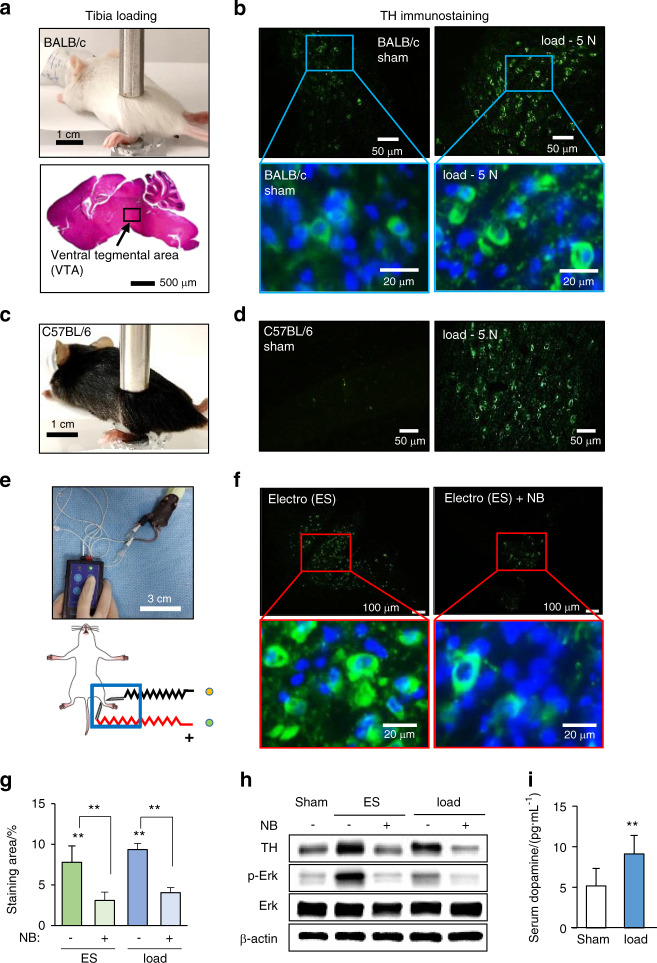
Elevation of tyrosine hydroxylase and dopamine by tibial loading. TH tyrosine hydroxylase, load tibial loading, ES electrostimulation, NB nerve block. The single and double asterisks indicate *P* < 0.05 and 0.01, respectively. **a** Setup for tibial loading with a BALB/c mouse. The ventral tegmental area (VTA) is shown in the sagittal section of a mouse brain. **b** Elevation of TH immunostaining in the VTA in the sagittal section of the loaded sample. **c**, **d** Load-driven elevation of TH in the VTA of C57BL/6 mice. **e**, **f** Elevation of TH induced by electrostimulation, and TH level suppression by local administration of lidocaine, a nerve block agent. **g** TH-immunostained area (in %) in the VTA induced by electrostimulation (ES) and tibial loading (load) showing that the application of a nerve block (NB) reduced the stained area. **h** Elevation of TH, p-Erk, and p-p38 in the brain induced by electrostimulation and tibial loading. The elevation of these factors was suppressed by the nerve block agent. **i** Elevation of dopamine in the serum by tibial loading. The serum was harvested 1 h after tibial loading

In response to mechanical loading (5 N at 2 Hz for 5 min) to the left and right tibiae, we observed that TH, a rate-limiting enzyme for dopamine synthesis, was elevated in the VTA in the brain of BALB/c mice 1 h after loading (Fig. 1a, b). Load-driven upregulation of TH was also detected in the VTA of C57BL/6 mice (Fig. 1c, d). Consistent with the elevation of TH, immunohistochemical analysis showed an increase in dopamine in the brain (Supplementary Fig. 1a). Notably, electrostimulation also elevated the TH level in the VTA (Fig. 1e, f), and the local application of lidocaine, a nerve block, abolished the upregulation of TH by electrostimulation and tibial loading (Fig. 1g). A Western blot analysis revealed that tibial loading elevated the level of TH in the brain, as well as the phosphorylated forms of Erk and p38 (p-Erk and p-p38), by both electrostimulation and loading to the tibia (Fig. 1h and Supplementary Fig. 1b). Notably, the upregulation of these factors was suppressed by the application of nerve block. ELISA quantification showed that the serum level of dopamine was increased by tibial loading (Fig. 1i). Collectively, the results indicated that dopamine in the brain can be elevated remotely by mechanical loading to the tibia.

### Dopamine inhibited the proliferation, migration, and invasion of mammary tumor cells

**Fig. 2:**
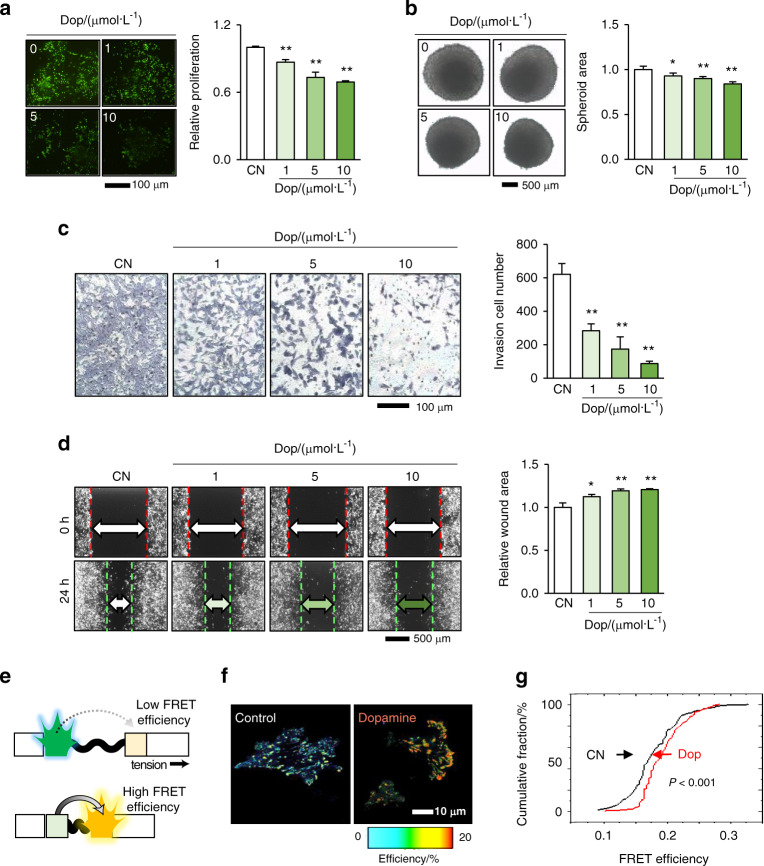
Inhibitory effects of dopamine in the proliferation, invasion, and migration of 4T1Br tumor cells. CN control, Dop dopamine. The single and double asterisks indicate *P* < 0.05 and 0.01, respectively. **a**, **b** Reduction in EdU-measured proliferation and the size of tumor spheroids upon exposure to dopamine. **c**, **d** Reduction in invasion determined by the Transwell assay and migration determined by the scratch assay after dopamine treatment. **e** FRET-based measurement of molecular forces. A FRET tension sensor is designed to increase FRET efficiency when the applied molecular force is reduced. **f**, **g** Dopamine-induced reduction in molecular forces with an increase in FRET efficiency

Since tibial loading was shown to elevate the level of dopamine in the brain and serum, we next examined the effect of dopamine on the cellular behaviors of 4T1Br brain-metastasized tumor cells. An EdU assay revealed that dopamine inhibited proliferation in a dose-dependent manner (Fig. 2a) and inhibited the growth of tumor spheroids (Fig. 2b). Dopamine also decreased Matrigel-based cellular invasion (Fig. 2c) and inhibited cellular motility in a scratch migration assay (Fig. 2d). The inhibitory action of dopamine was observed in 4T1.2 and MDA-MB-231 cells, which were isolated from metastatic bone and human-breast-cancer tissue, respectively (Supplementary Fig. 2). In a vinculin-based fluorescence resonance energy transfer (FRET) analysis performed to evaluate molecular forces in live-cell imaging, exposure of 4T1Br cells to dopamine elevated FRET efficiency in the vinculin biosensor and reduced molecular tension forces (Fig. 2e–g). The reduction in the molecular forces of the vinculin biosensor at focal adhesions was consistent with the inhibitory action of dopamine on cellular motility.

### Fluphenazine (FP) inhibited the proliferation, migration, and invasion of tumor cells

**Fig. 3:**
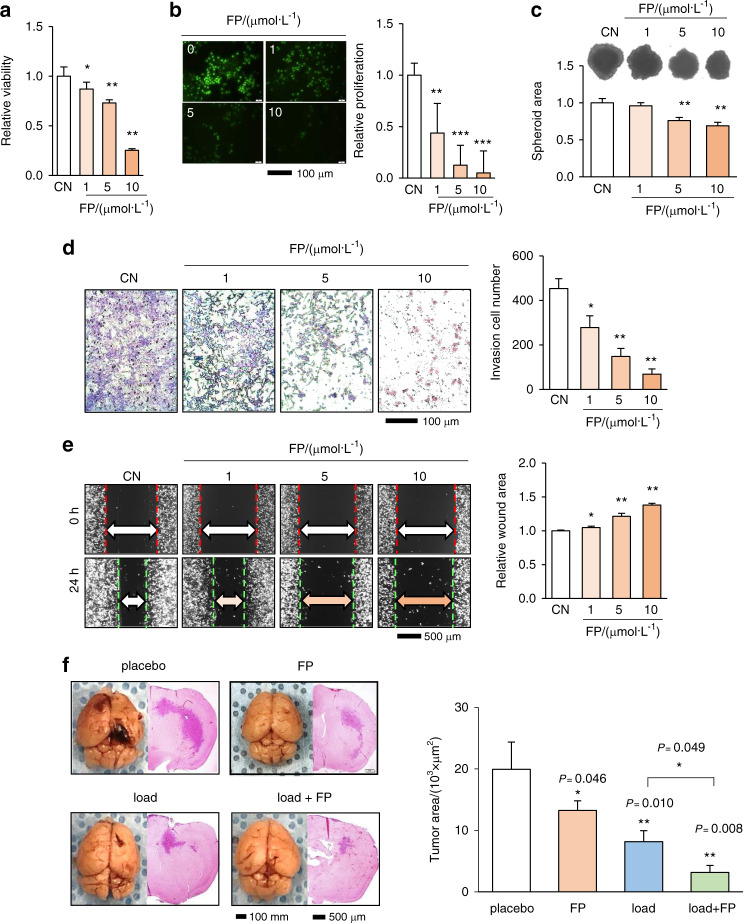
Inhibitory effects of fluphenazine (FP) on tumorigenic behaviors of 4T1Br cells. CN control, FP fluphenazine, pl placebo, load tibial loading. The single and double asterisks indicate *P* < 0.05 and 0.01, respectively. **a**–**c** Reduction in MTT-assessed viability, EdU-measured proliferation, and the size of tumor spheroids induced by FP. **d**, **e** Decrease in the Transwell invasion and scratch assay migration after exposure to FP. **f** Reduction in the tumor area in the brain of BALB/c mice by daily tibial loading with and without FP administration. The whole-brain image and the H&E-stained coronal sections, including the maximum tumor area. The maximum tumor area in the coronal section was quantified, including the placebo and the tibia-loaded groups with and without FP administration (*N* = 6 per group)

We previously reported that the FDA-approved dopamine modulator FP can reduce tumor-induced osteolysis in a mouse model.^25^ In addition to the tumor-suppressing effect of dopamine, we next examined the effect of FP on the tumorigenic behaviors of 4T1Br cells. The results revealed that FP also acted as a tumor-suppressing agent by reducing MTT-assessed cellular viability, EdU-measured proliferation, and the growth of tumor spheroids (Fig. 3a–c). It also reduced invasion as measured in Matrigel and motility as measured by the scratch assay (Fig. 3d, e).

### Tumor progression in the brain was markedly reduced by tibial loading and FP

To date, the administration of dopamine and FP has been shown to inhibit the proliferative and invasive behaviors of 4T1Br cells. Employing a mouse model of breast-cancer-associated brain tumors, we next examined the potential therapeutic efficacy of tibial loading and FP. Using BALB/c mice, FP was administered by intraperitoneal injection every other day for 2 weeks, and tibial loading (5 N, 2 Hz, 5 min) was applied daily.^3^ Histological analysis of the four groups (placebo, FP, loading, and loading with FP) revealed that the administration of FP significantly reduced tumor size in the brain (Fig. 3f). Furthermore, the tibial loading group showed a markedly reduced tumor-expansion area in the brain, and the simultaneous application of tibial loading and FP led to a significantly higher efficacy in reducing tumor growth in the brain (Fig. 3f).

### Dopamine receptor D1 mediated tumor suppression by loading-driven dopamine

**Fig. 4:**
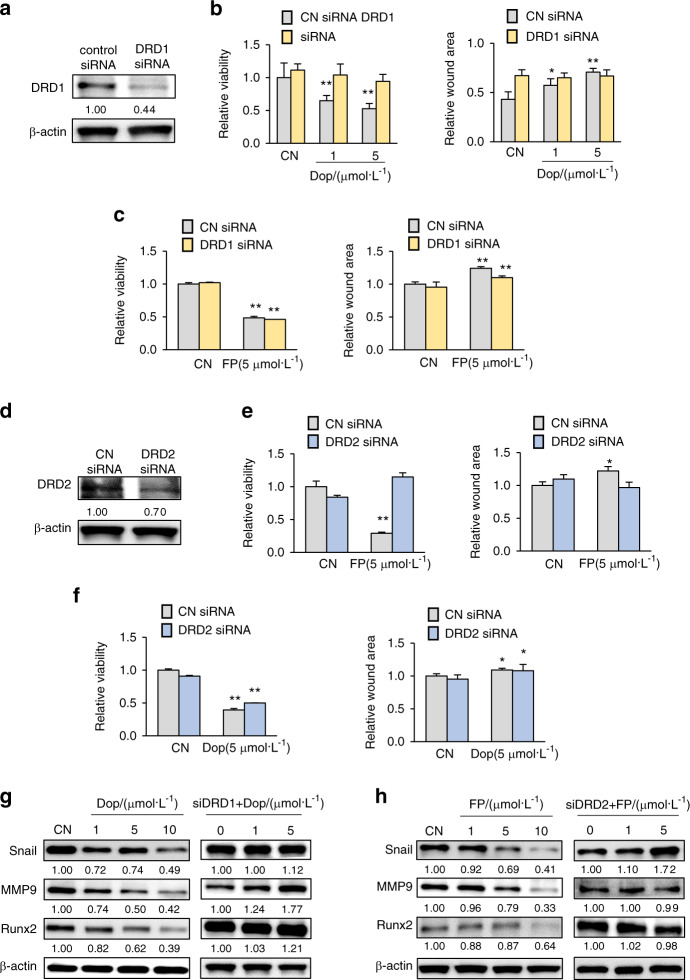
Role of two dopamine receptors (DRD1 and DRD2) in the responses to dopamine and fluphenazine in 4T1Br cells. CN control, Dop dopamine, FP fluphenazine, siDRD1 DRD1 siRNA, siDRD2 DRD2 siRNA. The single and double asterisks indicate *P* < 0.05 and 0.01, respectively. **a**–**c** Suppression of dopamine-driven inhibition of MTT-assessed viability and scratch assay-measured migration upon the silencing of DRD1. The inhibitory effect of FP was not altered by DRD1 silencing. **d**–**f** Suppression of the FP-driven inhibition of MTT-assessed viability and scratch assay-measured migration upon the silencing of DRD2. The inhibitory effect of dopamine was not altered by DRD2 silencing. **g** Dopamine-driven downregulation of Snail, MMP9, and Runx2, and the abolishment of their downregulation by DRD1 silencing. **h** FP-driven downregulation of Snail, MMP9, and Runx2, and the abolishment of their downregulation by DRD2 silencing

There are two types of dopamine receptors, types D1 and D2, among five known dopamine receptors (DRD1 to DRD5). To determine the tumor-suppressing mechanism induced by loading-driven dopamine increases, we focused on two dopamine receptors, DRD1 and DRD2, as representatives of types D1 and D2, respectively. In DRD1-silenced 4T1Br cells (Fig. 4a), dopamine-induced inhibition of viability and migration was abolished (Fig. 4b). However, FP-induced inhibition was not significantly altered (Fig. 4c). In contrast, in DRD2-silenced 4T1Br cells, the results of treatment was the opposite. Silencing DRD2 eradicated FP-induced inhibition but not dopamine-induced inhibition (Fig. 4d–f). In addition to tumorigenic behaviors, the downregulation of Snail, MMP9, and Runx2 by dopamine and FP was suppressed by the silencing of DRD1 and DRD2, respectively (Fig. 4g, h). Notably, the downregulation of Snail, Runx2, and TGFβ by dopamine was observed in three sources of primary human-breast-cancer cells (Supplementary Fig. 3). Collectively, the results support the notion that the antitumor actions of loading-driven dopamine are mediated by a type D1 receptor(s), and the action of FP is mediated by a type D2 receptor(s).

### Dopamine downregulated Lrp5 via DRD1

**Fig. 5:**
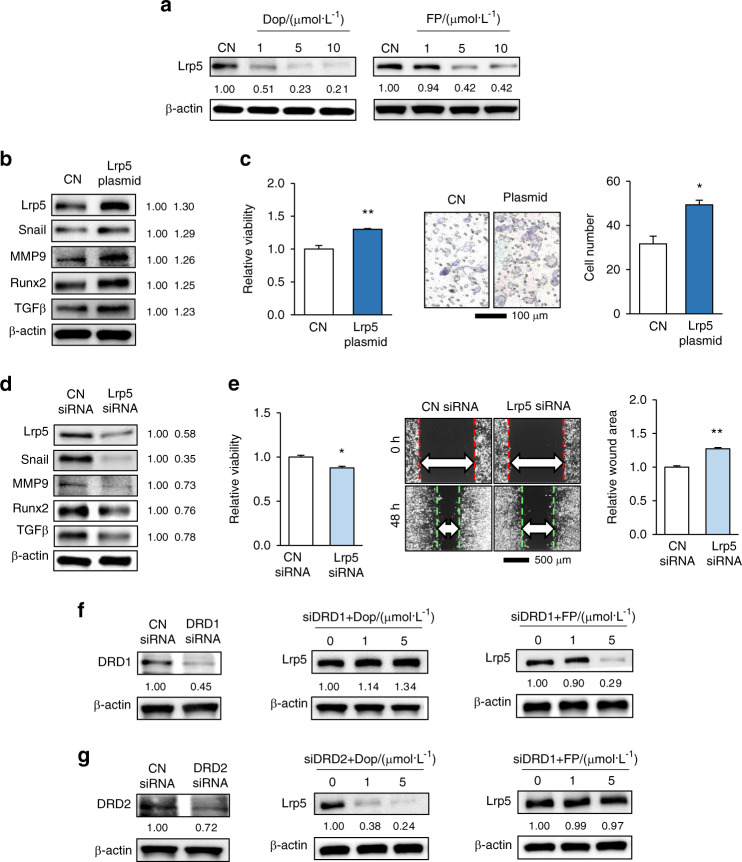
Downregulation of Lrp5 by dopamine and fluphenazine in 4T1Br cells. CN control, Dop dopamine, FP fluphenazine, siDRD1 DRD1 siRNA, siDRD2 DRD2 siRNA. The single and double asterisks indicate *P* < 0.05 and 0.01, respectively. **a** Reduction in Lrp5 by dopamine and fluphenazine. **b** Elevation of Snail, MMP9, Runx2, and TGFβ upon the overexpression of Lrp5. **c** Promotion of MTT-assessed viability and Transwell-measured invasion by the overexpression of Lrp5. **d** Reduction in Snail, MMP9, Runx2, and TGFβ levels by the silencing of Lrp5. **e** Decrease in MTT-assessed viability and scratch assay-measured migration by the silencing of Lrp5. **f** Suppression of dopamine-driven downregulation of Lrp5 by the silencing of DRD1. FP-driven downregulation was not suppressed. **g** Suppression of FP-driven downregulation of Lrp5 by the silencing of DRD2. Dopamine-driven downregulation was not suppressed

Wnt signaling is generally considered to promote tumor progression; however, it also plays an important role in loading-driven bone remodeling. As a coreceptor of Wnt signaling, Lrp5 in tumor cells may be regulated by dopamine to induce tumor-suppressing capabilities. Therefore, we first observed that Lrp5 was downregulated by dopamine and FP in tumor cells (Fig. 5a). In addition, the overexpression of Lrp5 elevated Snail, MMP9, Runx2, and TGFβ (Fig. 5b) and promoted the proliferation and invasion of 4T1Br tumor cells (Fig. 5c). In contrast, the silencing of Lrp5 reduced Snail, MMP9, Runx2, and TGFβ levels (Fig. 5d), as well as MTT-assessed viability and scratch assay-measured migration (Fig. 5e). In DRD1-silenced 4T1Br cells, dopamine, but not FP, was unable to reduce the level of Lrp5 (Fig. 5f). In DRD2-silenced 4T1Br cells, however, dopamine lowered the level of Lrp5 but not FP (Fig. 5g). Collectively, the results indicated that dopamine exerted antitumor effects by downregulating Lrp5 via the DRD1 receptor and FP via the DRD2 receptor.

### Mechanical loading and dopamine downregulated CCN4

**Fig. 6:**
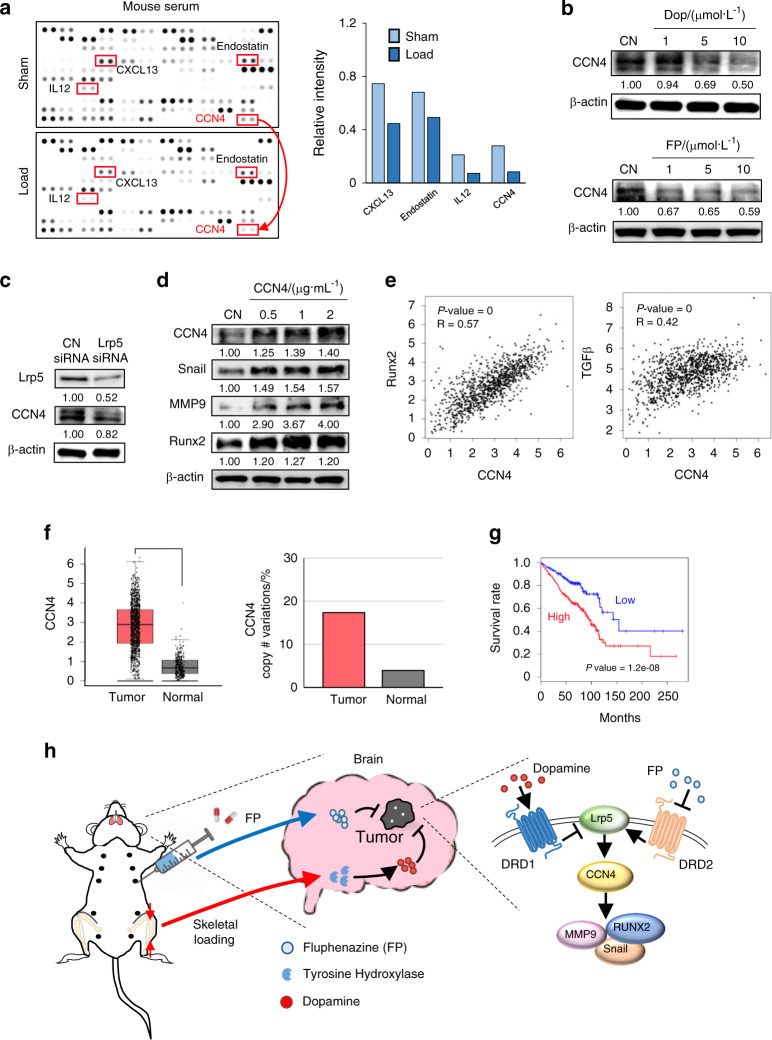
Tumor-promoting role of CCN4 and its downregulation by tibial loading. Notably, load tibial loading, CN control, Dop dopamine, FP fluphenazine. **a** Cytokine array analysis using the serum proteins obtained from tumor-inoculated BALB/c mice showing the downregulation of CXCL13, endostatin, IL12, and CCN4 by tibial loading. **b** Reduction in CCN4 level by dopamine and FP in 4T1Br cells. **c** Downregulation of CCN4 by the silencing of Lrp5. **d** Increase in Snail, MMP9, and Runx2 by the administration of recombinant CCN4 proteins in 4T1Br cells. **e** Transcript level correlation between CCN4/Runx2 and CCN4/TGFβ in patients with breast cancer in the TCGA database. **f** Elevation of CCN4 transcripts in breast-cancer tissues and the increase in CCN4 gene copy number. **g** Reduced survival rates of patients (with breast, lung, or prostate cancer) with high levels of CCN4 transcripts. **h** Schematic illustration of the regulatory mechanism induced through the dopamine-Lrp5 axis. In this model, tibial loading and FP chiefly act on DRD1 and DRD2, respectively, and they downregulate Lrp5, CCN4, MMP9, Runx2, and Snail

We thus far showed that dopamine and FP can act as tumor suppressors by blocking Lrp5-mediated Wnt signaling. The next step was the determination of the potential downstream effector, which links loading-driven regulation of the dopamine-Lrp5 axis to tumor-promoting genes such as Snail, MMP9, and Runx2. Since mechanical loading elevated the serum level of dopamine, a protein array analysis including 111 cytokines and proteins isolated from the serum was performed. Among four loading-responsive candidates (CXCL13, IL12, endostatin, and CCN4) (Fig. 6a), we selected CCN4, which is known as an oncogene inducible by Wnt signaling.^24^ A Western blot analysis with 4T1Br cells revealed that CCN4 was downregulated by dopamine and FP in a dose-dependent fashion (Fig. 6b). Furthermore, CCN4 was decreased in Lrp5 siRNA-treated 4T1Br cells (Fig. 6c), and incubation with recombinant CCN4 proteins elevated Snail, MMP9, and Runx2 (Fig. 6d). These results support the notion that CCN4 is a dopamine/Lrp5-responsive downstream regulator that is downregulated in the serum by mechanical loading.

Immunohistochemical analysis indicated the elevation of the DRD1 level in the brain of the loading group and the DRD2 level in the loading and FP groups, while the reduction of the levels of CCN4 and Lrp5 in these two groups was detected (Supplementary Fig. 4a). The Western blotting results using the protein samples isolated from the tumor-invaded brains were consistent with the immunohistochemical results (Supplementary Fig. 4b). We also detected the expression levels of DRD1 in four cell lines (4T1Br, 4T1.2, MDA-MB-231, and EO771 mammary tumor cells). EO771 cells showed a lower expression level of DRD1 than the three other cell lines (Supplementary Fig. 5a–d), and they presented weaker responsiveness to dopamine (Supplementary Fig. 5e–g). In summary, the result is consistent with the primary role of DRD1 in the dopamine response.

### A high level of CCN4 transcripts is linked to poor prognosis

To further understand the role of CCN4, we employed the Gene Expression Profiling Interactive Analysis (GEPIA) server and conducted genomic data analysis using the TCGA and Genotype-Tissue Expression (GTEx) databases.^26^ The level of CCN4 transcripts was positively correlated with Runx2 and TGFβ in tumor tissues from patients with breast cancer (Fig. 6e). Furthermore, in addition to the transcript level, the copy number of the CCN4 gene was elevated in tumor tissues (Fig. 6f). Most importantly, the survival rate for patients with a high level of CCN4 transcripts was markedly lower than that for patients with a low level of CCN4 transcripts (*P* = 1.2 × 10^−8^) among solid tumors (breast, lung, and prostate cancer patients) that preferentially metastasize to the brain (Fig. 6g).

## Discussion

This study presented the tumor-suppressing effects of remotely applied mechanical loading using a mouse model of breast-cancer-associated brain tumors. In response to tibial loading, the levels of TH and dopamine were elevated in the brain and the serum, respectively, and loading-driven regulation of dopamine synthesis in the brain was associated with the inhibition of the growth of brain tumors. Furthermore, simultaneous administration of FP strikingly reduced the size of brain tumors. Notably, FP is an FDA-approved antipsychotic drug used for the treatment of schizophrenia and psychotic symptoms. Because of the blood–brain barrier, dopamine cannot be delivered to the brain by standard intravenous injection. Loading-driven dopamine downregulated Lrp5 in 4T1Br brain-metastasized tumor cells via dopamine receptor D1, followed by a reduction in CCN4, an oncogene inducible by Wnt signaling. Although Lrp5 is indispensable for loading-driven bone formation, its downregulation was critical to tumor suppression. Collectively, remote mechanotransduction from the bone to the brain provided the possibility of treating brain tumors noninvasively by regulating the dopamine-Lrp5-CCN4 axis.

Many studies have shown that mechanical loading to bone induces both local and global effects.^27,28^ We have previously shown that loading to the mouse hind limb raised the amount of serotonin synthesized by activating tryptophan hydroxylase, a rate-limiting enzyme for serotonin production.^29^ The results of this study showed that dopamine synthesis is also elevated in the brain by TH, and this increase is followed by an increase in the serum dopamine level. While dopamine in the present study was induced by mechanical loading, it has been reported that electrostimulation can also elevate dopamine.^30^ The loading-driven elevation of dopamine was suppressed by the application of a local anesthetic, such as lidocaine, indicating that the load-driven upregulation in dopamine is mediated by neuronal signaling. The results showed that TH expression in the brain was upregulated by electrostimulation and loading, and this upregulation was suppressed by a nerve block. Furthermore, the regulation of p-Erk and p-p38 was associated with alterations in TH levels. It has been reported that Erk is involved in the regulation of TH and the synthesis of dopamine in the brain.^30^ Further analysis is recommended to establish the mechanism of the effect of loading on Erk and p38 via neuronal signaling.

In the regulatory mechanism (Fig. 6h), loading-driven dopamine exerts its tumor-suppressing effect via DRD1, while the effects of FP are mediated via DRD2. The activation of DRD1 was reported to inhibit the growth of glioblastoma,^31^ and in the same study, tumor cells with a high DRD1 level were more sensitive to dopamine than those with a low level. The results from the present study indicate that the efficacy of mechanical loading and FP in cancer cells may depend on the expression levels of DRD1 and DRD2. Lrp5 is a coreceptor of Wnt signaling and is known to play a crucial role in the mechanotransduction of bone^22^ and an undesirable role in tumor progression.^32^ The reduction in Lrp5 via dopaminergic signaling inhibited CCN4, followed by the downregulation of MMP9, Runx2, Snail, and TGFβ.^19^ CCN4 is known to stimulate tumor growth via Wnt signaling, and its downregulation through loading-driven inhibition of Lrp5 is essential for tumor suppression. Analyses using the TCGA and GTEx databases revealed that the overall survival rate of patients with a high level of CCN4 transcripts was markedly reduced. Taken together, the results support the pivotal role of the dopamine-Lrp5-CCN4 axis in loading-driven tumor suppression.

The majority of current treatments for brain metastasis are palliative and destructive, with a limited benefit for survival.^33^ In contrast to most chemotherapeutic agents that fail to penetrate the blood–brain barrier, FP, an FDA-approved anti-schizophrenia drug, can enter the brain. VOC analysis revealed that skeletal loading has a role in the regulation of cholesterol (Supplementary Fig. 6), which is consistent with our previous clinical work on step aerobics.^16^ In this study, we have shown that the urinary level of dopamine is elevated by step aerobics. Collectively, we postulate that further VOC analysis of urine may lead to novel diagnostic markers or therapeutic targets. While the results herein suggest the novel possibility of treating breast-cancer-associated brain metastasis via mechanical loading, the study has limitations. Our experiments used a mouse brain-metastasized tumor cell line and breast-cancer cell lines. The dopamine response depends on the expression level of dopamine receptors, and the efficacy may depend on cancer cell types. In addition, the loading conditions are largely different in preclinical studies with small animals and clinical applications. The proper loading force may depend on its in situ or remote use. We employed a 5-N force to induce remote tumor-suppressing effects in this study, whereas the previous study for suppressing tumor progression in the loaded tibia employed a 1-N force.^3^ Based on the difference in the diameter of the mouse and human tibiae in the midshaft (1.3 mm vs. 22 mm), the cross-sectional area differs by ~300-fold, and 5-N loads to mice may correspond to 1 500 N to humans. The internal loads to the tibia in humans are predicted to be as high as 4.7 bodyweights.^34^ For a person with a body weight of 65 kg, the maximum load is ~3 000 N. The required loads are not proportional to bodyweights across species, and further analysis is needed to predict the equivalent loads applicable to humans.

In summary, this study revealed that loading-driven dopamine regulation induced a markedly effective, noninvasive antitumor effect. This study is the first to show that the regulation of the dopamine-driven regulation of the Lrp5-CCN4 axis via the DRD1 receptor is central to remote mechanotransduction from the bone to the tumor-invaded brain. CCN4 is highly expressed in tumor tissues, and its high expression level significantly lowers the overall survival rate. The results herein suggest that the regulation of Lrp5-mediated dopaminergic signaling may provide a novel strategy to restrain breast-cancer-associated tumor growth in the brain.

## Materials and methods

### Cell culture

4T1Br,^35^ 4T1.2 mammary tumor cells (obtained from Dr. R. Anderson at Peter MacCallum Cancer Institute, Australia),^36^ EO771 mammary tumor cells (CH3 BioSystems, Amherst, NY, USA),^37^ and MDA-MB-231 human-breast-cancer cells^38^ were grown in DMEM (Corning, Inc., Corning, NY, USA). Notably, the 4T1Br cell line is a clone of the 4T1 cell line derived from metastasized brain. The culture medium was supplemented with 10% fetal bovine serum and antibiotics (100 U·mL^−1^ penicillin and 100 μg·mL^−1^ streptomycin; Gibco, Grand Island, NY, USA). Primary human-breast-cancer cells were grown as described previously.^39^ The cells were maintained at 37 °C with 5% CO_2_. Tumor cells were treated with dopamine (Tocris, Minneapolis, MN, USA), fluphenazine (FP; Sigma, St. Louis, MO, USA), or CCN4 recombinant proteins (BioLegend, San Diego, CA, USA).

### MTT, EdU, scratch, and invasion assays

Cell proliferation was examined by MTT and a fluorescence-based EdU cell proliferation kit (Click-iT™ EdU Alexa Fluor™ 488 Imaging Kit; Thermo-Fisher, Waltham, MA, USA) as previously described.^25^ A wound-healing scratch assay and an invasion assay were conducted to evaluate cell motility and invasion capability using procedures previously described.^25^

### Western blot analysis, ELISA, and protein array analysis

We used antibodies against Src, p-Src, Snail, TGFβ, Lrp5, Runx2 (Cell Signaling, Danvers, MA, USA), DRD_1_, DRD_2_ (Abcam, Cambridge, MA, USA), MMP9 (Santa Cruz Biotechnology, Dallas, Texas, USA), TH (Novus Biologicals, Centennial, CO, USA), CCN4 (R&D Systems, Minneapolis, MN, USA), and β-actin (Sigma). Protein levels were assayed using a SuperSignal West Femto maximum sensitivity substrate (Thermo-Fisher). The level of dopamine in the serum was determined using an ELISA kit (MyBioSource, San Diego, CA, USA). We also employed a mouse XL cytokine array (R&D Systems, Minneapolis, MN, USA) and determined the expression of 111 cytokines and chemokines in mouse serum samples.

### Spheroid assay, RNA interference, and plasmid transfection

For a spheroid assay, cells were cultured in a U-bottom low-adhesion 96-well plate (S-Bio, Hudson, NH, USA) in DMEM (10% FBS, 1% antibiotics). RNA interference was conducted using siRNA specific to DRD_1_, DRD_2_, and Lrp5 (Thermo-Fisher) with a negative siRNA (Silencer Select #1, Thermo-Fisher) using a procedure previously described.^25^ To overexpress Lrp5, Lrp5 plasmids (#115907, Addgene, Watertown, MA, USA) were transfected into 4T1Br cells with pcDNA used as a control plasmid.

### Fluorescence resonance energy transfer (FRET)

To evaluate tension forces at focal adhesions in the presence and absence of dopamine, a plasmid expressing a vinculin tension sensor (VinTS, #26019, Addgene) was transfected, and fluorescence lifetime images were acquired using procedures previously described with a custom-made microscope attached to a laser scanning confocal microscope (FluoView 1000, Olympus, Center Valley, PA, USA).^40^ Notably, an elevation in the tension force of the vinculin tension sensor implies a decrease in FRET efficiency.

### Animal model for brain tumors associated with breast cancer

The experimental procedures were approved by the Indiana University Animal Care and Use Committee and complied with the Guiding Principles in the Care and Use of Animals endorsed by the American Physiological Society. In the mouse model of breast-cancer-associated brain tumors, 52 BALB/c female mice (~8 weeks, Envigo, Indianapolis, IN, USA) were allocated to four groups: placebo, loading, FP injection with and without loading groups. The mice were assigned by a stratified randomization procedure based on body weight. Without drilling a burr hole in the skull, mice received an inoculation of 4T1Br cells (5 000 cells in 15 μL of PBS) into the right frontal lobe of the brain using a 29 G needle as previously reported.^41^ Instead of an intracardiac injection,^42^ the injection to the brain was selected to increase the success rate of tumor growth in the brain and reduce the number of mice needed. After 2 days of recovery, the mice received an i.p. injection of FP (3 mg·kg^−1^, every other day), underwent tibial loading (5 N at 5 Hz for 5 min daily) to the left and right limbs, or received a combination of FP and tibial loading. Twelve days after the inoculation of tumor cells, the whole brain was harvested for Western blot and histological analyses, urine was harvested for VOC analysis, and serum was harvested for ELISA.

### Tibial loading and electrostimulation

Tibial loading for tumor-inoculated mice was performed as previously described.^3^ Using an ElectroForce device (TA instruments, New Castle, DE, USA), loads were applied daily to the left and right tibiae of a mask-anesthetized mouse. Using 10 BALB/c mice and 10 C57BL/6 mice, electrostimulation was also applied with a commercially available device (MicroStim Plus peripheral nerve stimulator; Neurotech, Waukesha, WI, USA). In brief, two electric terminals were taped to the medial and lateral sides of the left knee of the anesthetized animal, and electric stimulation (10 mA at 1 Hz for 1 min) was applied. The whole brain was isolated 30 min after stimulation to evaluate the level of TH in the brain.

### Histology

Coronal brain sections were fixed in 4% paraformaldehyde in PBS. They were dehydrated through a series of graded alcohols, cleared in xylene, and embedded in paraffin. To determine the distribution of tumor cells, we conducted H&E staining and evaluated coronal sections at 60-μm intervals. The tumor area was quantified as the ratio of a lesion to the whole-brain area. Using immunohistochemical staining, we also detected the expression levels of TH, dopamine, DRD1, DRD2, CCN4, and Lrp5 in coronal sections. After deparaffinization, the sections were placed in 10-mM citric acid buffer for antigen activation. The sections were incubated with primary antibodies, followed by alkaline phosphatase-conjugated secondary antibody, which was used for chromogenic staining. The sections were counterstained with H&E and evaluated at 60-μm intervals. The immunostained area was quantified with respect to the tumor area. For immunohistochemistry, tumor cells were fixed in 4% paraformaldehyde in PBS. After the primary and secondary antibodies were stained, nuclei were stained with DAPI at room temperature for 20 min.^43^

### Evaluation of CCN4 in the TCGA data

Using the GEPIA server, we analyzed a patient-driven genomic TCGA dataset and data on normal tissues obtained from the GTEx project.^26^ We evaluated the potential role of CCN4 in cancer prognosis, focusing on the transcript level of tumor-promoting genes in tumor and normal tissues, the increase in the copy number of the CCN4 gene, and the overall survival rate of patients with low and high levels of CCN4 transcripts.

### Statistical analysis

For cell-based experiments, three or four independent experiments were conducted, and the data are expressed as the means ± S.D. Statistical significance was evaluated using one-way analysis of variance. Post hoc statistical comparisons with control groups were performed using Bonferroni’s correction with significance set to *P* < 0.05. A nonparametric Kolmogorov–Smirnov test was applied to evaluate molecular forces revealed through FRET imaging.

## Supplementary information

supplement figure and method
