# Supplementary material for: Mechanical tibial loading remotely suppresses brain tumors by dopamine-mediated downregulation of CCN4
Source: Bone Res. 2021 May 24;9:26. doi: 10.1038/s41413-021-00144-2 (PMC8144433; doi:10.1038/s41413-021-00144-2)
Supplement: Supplementary file 1 — supplement figure and method [file 41413_2021_144_MOESM1_ESM.docx]

**Supplementary Information**

**Supplementary Materials and Methods**

**Analysis of** **urinary volatile organic compounds (VOCs).** Using 12 BALB/c mice without tumor inoculation, 21 urine samples (50 μl) were collected (12 from the no-loading control group, and 9 from the loading group). The no-loading control samples were collected before tibia loading and the loading samples were collected 1 h after tibia loading. VOCs were analyzed using the procedure previously described (Woollam et al., 2019). A matrix with 226 VOCs was generated through the spectral alignment of sample chromatograms. Integrated signals were analyzed and the compounds statistically significant (*p* < 0.05) in the two groups were identified by a two-tailed Student’s t-test. The best three upregulated and best three downregulated VOCs (lowest p-values) were used for hierarchical clustering analysis and principal component analysis, using MATLAB (R2018b; Math Works, Natick, MA, USA). The NIST 14 mass spectral library was used to preliminarily identify differentially excreted molecular features. All three upregulated VOCs were analyzed by the gas chromatograph-mass spectrometer which confirmed compound identification by NIST. A hierarchical clastergram highlighted 6 representative VOCs (Supp. Fig. 5a&b). In the principal component analysis, the left cluster is the three VOCs diminished by loading, in which two of them are linked to the mevalonate pathway leading to cholesterol synthesis (Suppl. Fig. 5c). Taken together, urine analysis indicated that mechanical loading reduced cholesterol-linked VOCs.

**Supplementary Figures**

| 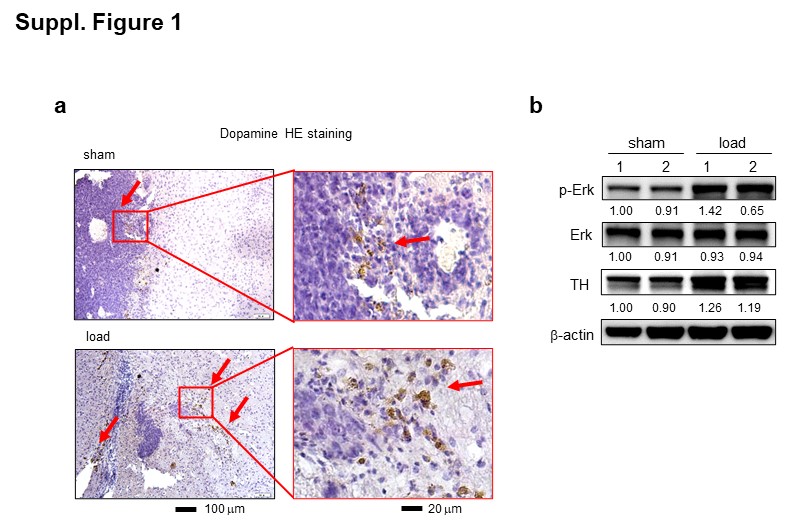 |
| --- |
| **Suppl. Fig. 1.** Dopamine expression and Erk-mediated regulation of tyrosine hydroxylase (TH). **a** Immunohistochemical analysis of dopamine in the ventral tegmental area of the brain. The left is the sham-loaded sample, while the right the tibia-loaded sample. The sections were H&E-stained, and the red arrows indicate the areas stained with dopamine (brown dots). **b** Elevation of phosphorylated Erk (p-Erk) and TH by tibia loading in the brain. **c** Reduction of p-Erk and TH by PD98059 (inhibitor of Erk) in GT1-7 mouse hypothalamic neuronal cells. |

| 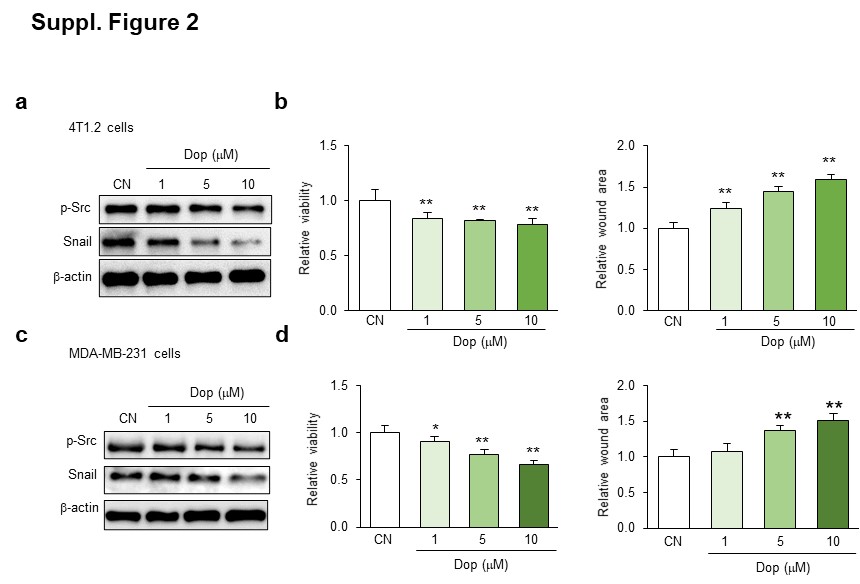 |
| --- |
| **Suppl. Fig. 2.** Suppression of tumorigenic behaviors of 4T1.2 and MDA-MB-231 cells in response to dopamine. CN = control, and Dop = dopamine. The single and double asterisks indicate *p* < 0.05 and 0.01, respectively. **a** Dopamine-driven downregulation of p-Src, and Snail in 4T1.2 cells. **b** Dopamine-driven reduction in MTT-based viability and scratch-based migration of 4T1.2 cells. **c** Dopamine-driven downregulation of p-Src and Snail in MDA-MB-231 cells. **d** Dopamine-driven reduction in MTT-based viability and scratch-based migration of MDA-MB-231 cells. |

| 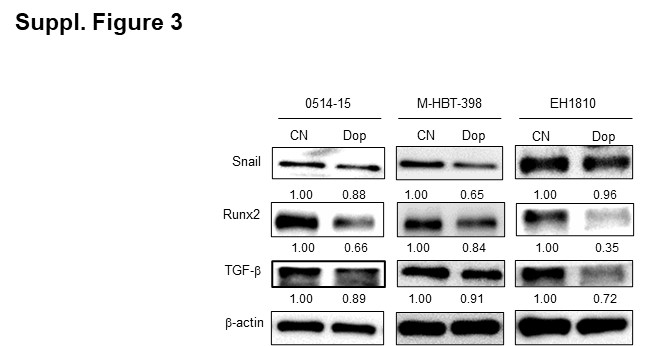 |
| --- |
| **Suppl. Fig. 3.** Reduction in the level of Snail, Runx2, and TGFβ by 10 μM dopamine in three sources of primary human breast cancer cells. |
| 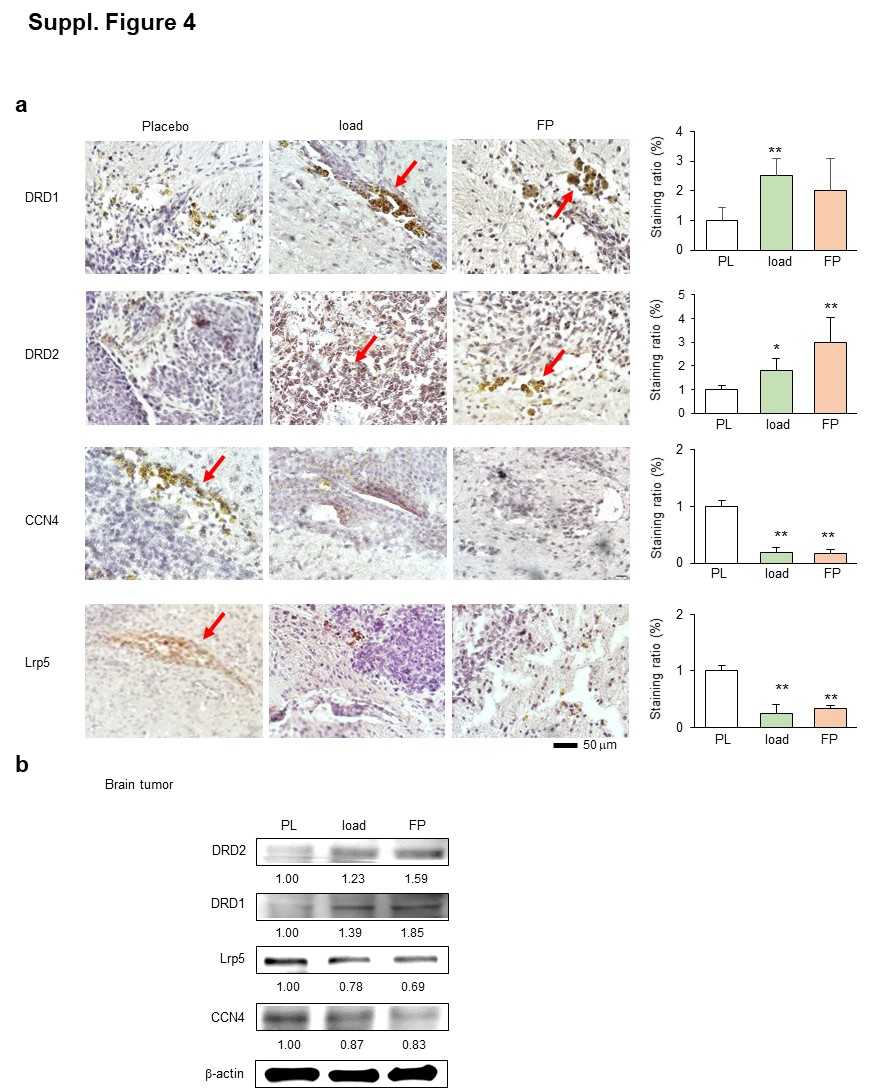 |
| **Suppl. Fig. 4.** Immunohistochemical and Western blot analyses of the expression of DRD1, DRD2, CCN4, and Lrp5 in tumor-invaded brains. PL = placebo, loading = tibia loading, and FP = Fluphenazine. The single and double asterisks indicate p < 0.05 and 0.01, respectively. **a** Immunohistochemical analysis of DRD1, DRD2, CCN4, and Lrp5 in the brain sections in the placebo, loading, and FP groups. The coronal sections in the midbrain were H&E-stained. The red arrows indicate the areas that were stained with the brown signal. The quantification was conducted to determine the ratio of the brown area to the tumor-invaded area. **b** Elevation of the levels of DRD1, and DRD2, as well as the reduction in the level of CCN4 and Lrp5 in the loading and FP groups. Proteins were isolated from the tumor-invaded brain. |
| 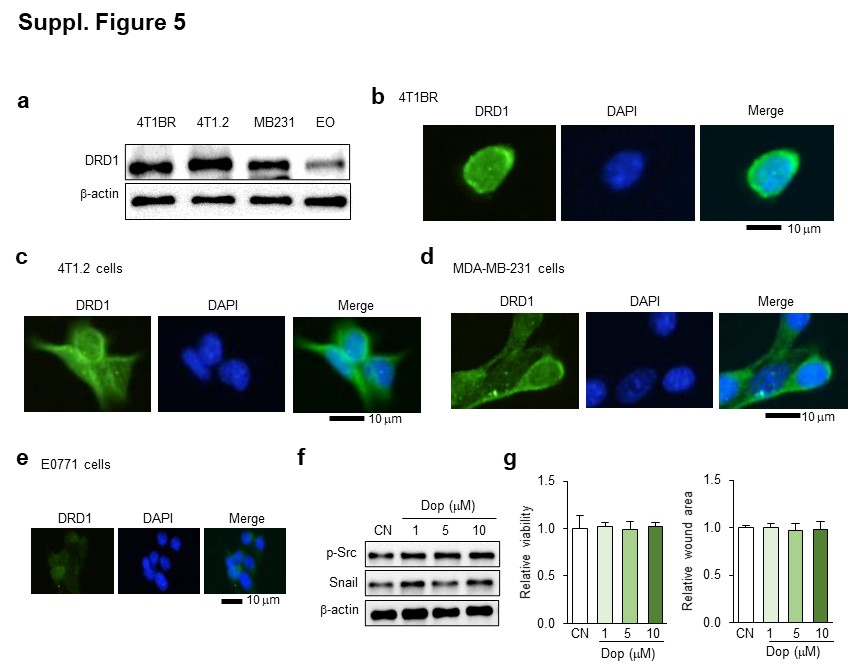 |
| **Suppl. Fig. 5.** Involvement of DRD1 in dopamine responses. The single and double asterisks indicate p < 0.05 and p < 0.01, respectively. Of note, CN = control, and Dop = dopamine. **a** Baseline DRD1 levels in 4T1Br, 4T1.2, MDA-MB-231, and EO771 cells. **b** Immunochemistry for DRD1 expression in 4T1Br cells. **c** Immunochemistry for DRD1 expression in 4T1.2 cells. **d** Immunochemistry for DRD1 expression in MDA-MB-231 cells. **e** Immunochemistry for DRD1 expression in EO771 cells. **f** Undetectable change in p-Src and Snail by dopamine in EO771 cells. **g** Undetectable change in MTT-based cell viability and cellular migration by dopamine in EO771 cells. |

| 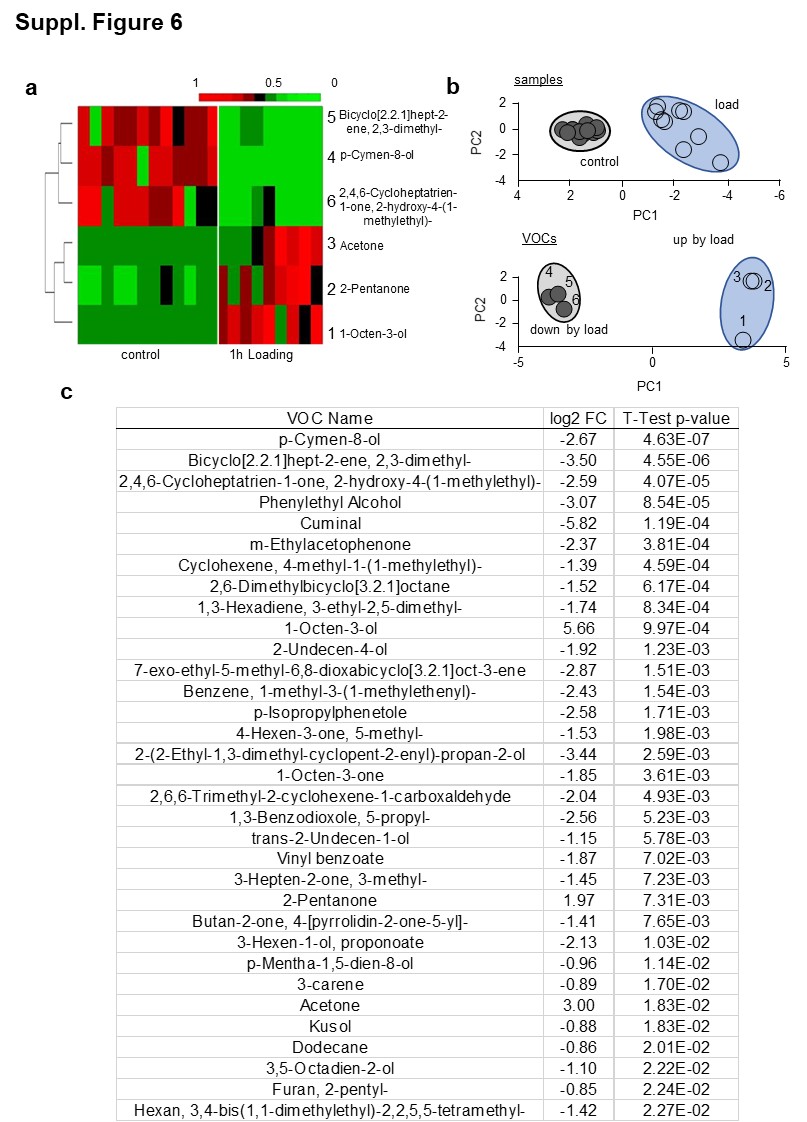 |
| --- |
| **Suppl. Fig. 6.** Analysis of urinary volatile organic compounds (VOCs) in response to tibia loading. **a** Hierarchical clustering of the control samples and tibia-loaded samples. The red and green colors indicate the high and low levels of VOCs, respectively. Six VOCs were highlighted to identify the differential signature in the control and tibia-loaded groups. **b** Two-dimensional principal component analysis that separated VOCs between the control and tibia-loaded groups. **c** List of 33 urinary VOCs, whose urinary levels were significantly altered by tibia loading. |
